# Physical Activity Prescriptions: Addressing a Major Gap in Medical Education

**DOI:** 10.1007/s40670-025-02382-z

**Published:** 2025-04-02

**Authors:** Timothy C. Frommeyer, Garrett V. Brittain, TongFan Wu, Daniel Frommeyer, Ean S. Bett

**Affiliations:** 1 Department of Internal Medicine, The Ohio State University Wexner Medical Center, 395 W 12Th Avenue, 3rd Floor, Columbus, OH USA; 2 Department of Family Medicine, The Ohio State University Wexner Medical Center, Columbus, OH USA; 3 University of Dayton, Dayton, OH USA; 4 Fairfield Medical Center, Lancaster, OH USA

**Keywords:** Exercise prescriptions, Exercise is medicine, Lifestyle medicine, Chronic disease, Medical education

## Abstract

In the United States and around the world, physical inactivity is a leading cause of morbidity, disability, and premature mortality. While the importance of physical activity is known, there is a disconnect in how we are translating it to our patients. This may be due to poor physical activity education during undergraduate and graduate medical education. Despite the paucity of physical activity prescription education and competence, there are ample resources within the community. It is time for physicians to take an active role in optimizing patient lifestyle and helping prevent or mitigate chronic diseases.

Physicians are committed to heal the sick and prevent disease. However, modern medicine is reactive in nature and fails to deter disease development. This is best evidenced by our failure to integrate one of the most efficacious treatments available for our patients: physical activity (PA).

I admitted a middle-aged female with an non-ST segment elevation myocardial infarction (NSTEMI). She had a high Body Mass Index (BMI) and lived a sedentary life, but recently started healthy eating to lose weight. The patient had many questions regarding the development of her disease, especially given her recent healthy lifestyle changes. How could she have a heart attack even though she was middle-aged, eating healthy, and losing weight? I explained modifiable versus non-modifiable risk factors and the chronicity of coronary artery disease (CAD). We also discussed PA as one of the best therapies in preventing the progression of CAD. She endorsed trying to start PA for years but was unsure where to begin. The patient had sought guidance from her primary care provider but did not receive recommendations or an effective plan outside the standard “30 min of daily exercise to meet at least 150 min a week”. Despite her motivation, she unfortunately did not know how to proceed.

This patient is not alone in suffering the consequences of inadequate PA and lack of guidance from healthcare providers. In the United States (US) and around the world, physical inactivity is a leading cause of morbidity, disability, and premature mortality. Despite the importance of PA, approximately 80% of US adults and adolescents are insufficiently active, adding to the growing epidemic of obesity and chronic disease[1]. PA may provide the greatest benefit of all the medications and therapies. An analysis of over 100,000 people over 30 years found that individuals who performed consistent PA had significantly lower all-cause mortality (ACM) (Figs. 1) [2]. Participants who met the US Department of Health and Human Services’ recommended 150–300 min/week of moderate PA had an observed 20–21% reduced ACM. Individuals who performed two to four times the recommended amount of PA were observed to have further declines in mortality. There were also reductions in cardiovascular disease (CVD) mortality by almost 40%. No harmful cardiovascular health effects were found among the study population. These findings align with others in the medical literature, supporting PA as a highly effective and safe therapy that significantly improves CVD mortality and ACM[3].

**Fig. 1:**
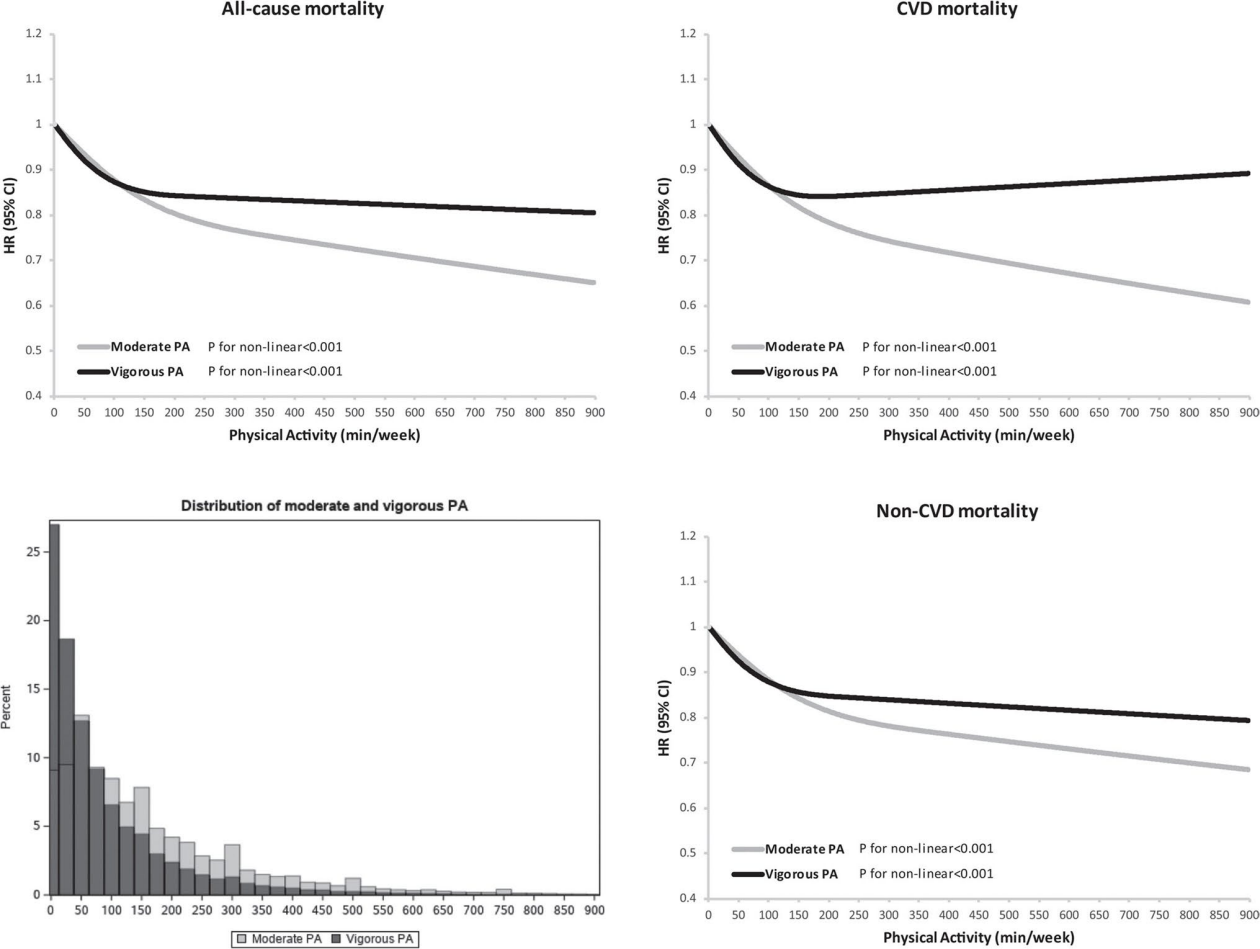
Dose–response relationship of long-term leisure-time moderate physical activity and vigorous physical activity with all-cause mortality. Reproduced from: D. Lee et al. “Long-Term Leisure-Time Physical Activity Intensity and All-Cause and Cause-Specific Mortality: A Prospective Cohort of US Adults.” Circulation. 2022 Aug 16;146(7):523–534.^2^ https://www.ahajournals.org/doi/. The Creative Commons license does not apply to this content. Use of the material in any format is prohibited without written permission from the publisher, Wolters Kluwer Health, Inc. Please contact permissions@lww.com for further information

While the importance of PA is known, there is a disconnect in how we are translating it to our patients. This disparity may be a result of insufficient training in PA prescription. In 2001, a national survey found only 10% of graduating medical students could recommend an exercise prescription[4]. This finding is unsurprising given only 13% of medical schools in the United States (US) provided an exercise curriculum in 2002[5]. However, nearly 20 years later, only 10% of medical students received formal exercise education and less than half are confident in creating an exercise routine for patients[6]. We are taught to diagnose and manage various pathologies, typically with medications or procedures, yet ineffective at guiding our patients to stay healthy through consistent PA. This may be due to poor PA education during undergraduate and graduate medical education.

Despite the paucity of PA prescription education and competence, there are ample resources within the community. Exercise is Medicine (EIM) was founded in 2007 by the American College of Sports Medicine to make PA a standard part of disease prevention and the treatment model for patient care[7]. Their initiative offers healthcare providers and patients with the resources to effectively optimize PA for those under our care[8]. The medical community needs to become leaders in motivating and providing the tools necessary for patients to start PA. As such, undergraduate and graduate medical education must similarly prioritize PA as medicine and integrate relevant teachings, such as those from EIM, within standard curriculum. The rapidly declining health of Americans calls for our healthcare system to place importance on lifestyle changes to offer the most optimal patient outcomes. This change starts within the medical education of students, residents, and practicing physicians.

Curriculum and its workload is extremely saturated in both undergraduate and graduate medical education. To maximize the benefits of EIM curriculum, its integration must also be efficient in order to minimize altering other vital aspects of the educational program. To address this, the American Medical Society for Sports Medicine (AMSSM) formed a group to develop curricular guidance for exercise medicine and physical activity prescriptions for medical school, residency, and fellowship levels of training[9]. Using a modified Delphi process, a group of sports and exercise medicine (SEM) experts created three training level-specific curricula regarding EIM, which was further reviewed by further SEM experts, fellowship directors, and the AMSSM board of directors (Tables 1 & 2). This proposed curriculum could be utilized broadly by medical schools, residencies, and fellowships to address the major deficit in prescribing PA. Its competencies, learning areas, and practical elements (how the curriculum is being learned) could be supplemented within standard curriculum without increasing workload or removing other curricular objectives. It is time for physicians to take an active role in optimizing patient lifestyle and helping prevent or mitigate chronic diseases.

**Table 1:** Medical school education curriculum

Domain	General learning areas	Specific learning areas
1. Foundations of Exercise Medicine and Physical Activity Promotion	1.1 Understand the basics of exercise in health and medicine	1.1.1 Describe the role of exercise medicine and physical activity in disease prevention/treatment 1.1.2 Review standard recommendations for exercise, physical activity, step counts, and sedentary behaviour in the general population, including children, teenagers, and adults.17 18 1.1.3 Outline the FITT (frequency, intensity, time, and type) or FITTvolume, progression framework
	1.2 Understand the basics of exercise prescription	1.2.1 Review general principles of writing an exercise prescription 1.2.2 Write a general exercise prescription 1.2.3 Outline safety considerations/contraindications
	1.3 Understand the basics of behavioural change	1.3.1 Define models of behaviour change 1.3.2 Review the practical aspects of motivational interviewing and brief intervention that can be used in the clinical setting
2. Exercise Prescriptions in Medical Conditions and Special Populations	2.1 Learn modifications and recommendations for exercise in various common conditions (may be incorporated into case-based discussions)	2.1.2: Pulmonary: COPD, asthma, and cystic fibrosis 2.1.3 Gastrointestinal: post-abdominal surgery 2.1.4 Renal: hypertension 2.1.5 Neurosciences: depression 2.1.6 Musculoskeletal: knee osteoarthritis, low back pain, physical disability 2.1.7 Endocrine: type II diabetes 2.1.8 Reproductive: pregnancy 2.1.9 Haematology-oncology: cancer, sickle cell trait/disease
3. Exercise Medicine and Physical Activity Promotion in Clinical Practice	3.1 Experience writing exercise prescriptions during patient encounters	3.1.1 Assess physical activity levels during ambulatory patient encounters 3.1.2 Write exercise prescriptions as part of ambulatory clinical rotations
	3.2 Promote individual and community engagement	3.2.1 Learn general barriers (including social and economic) to physical activity that are specific to an individual 3.2.2 Learn general barriers (including the built environment, traffic, pollution, and climate) to physical activity that are specific to a community 3.2.3 Identify community resources available for patients to engage in physical activity 3.2.4 Learn that bias (implicit or explicit) can affect one’s ability to counsel patients
Practical elements		
• Assess physical activity levels and write exercise prescriptions for five patients as part of an ambulatory clinical rotation (eg, Family Medicine Clerkship/rotation*) • Assess physical activity levels and write exercise prescriptions for 10 patients as part of an ambulatory acting internship/subinternship.† • Locate at least three different types of accessible resources (eg, community partners, online videos) that can be used to assist physical activity promotion for patients or a community • Identify at least one funding source to assist patients in need of financial assistance to conduct a physical activity programme • Participate in a local fitness event (charity run/walk, group fitness class, etc.)		

*The introductory rotation in family medicine and preventative care

†An ambulatory acting internship/subinternship is an advanced level of clinical training beyond the basic clerkship which provides an opportunity for greater autonomy and care for patients in a way that approximates early postgraduate (internship) training.

Reproduced from: L. Asif et al. “Exercise medicine and physical activity promotion: core curricula for US medical schools, residencies and sports medicine fellowships: developed by the American Medical Society for Sports Medicine and endorsed by the Canadian Academy of Sport and Exercise.” British Journal of Sports Medicine. 2022 Apr;56(7):369–375.^9^ https://bjsm.bmj.com/content/56/7/369—with permission from the BMJ Publishing Group Ltd

**Table 2:** Residency education curriculum

Domain	General learning areas	Specific learning areas
1. Healthy Bbehaviours	1.1 Identify the role of physical activity, nutrition, mental health, sleep, and reduction of substance use in health promotion and disease prevention	1.1.1 Examine how physical activity—as part of a broader curriculum focused on healthy behaviours—can be used to prevent/treat common diseases encountered within a residency, across disciplines/specialties 1.1.2 Demonstrate motivational interviewing techniques that could be used to promote healthy behaviours 1.1.3 Demonstrate ways to work with patients who disagree or cannot complete recommendations
Practical elements		
Use motivational interviewing techniques to promote healthy behaviours with 10 patients		
2.Exercise Medicine and Physical Activity Promotion in Clinical Practice	2.1 Practice physical activity promotion and exercise counselling during patient encounters	2.1.1 If needed, review the Medical School Curriculum in Exercise Medicine and Physical Activity Promotion, including disease-specific exercise recommendations and motivational interviewing best practices in the clinical setting 2.1.2 Implement routine assessment of physical activity levels of patients during ambulatory clinical patient encounters 2.1.3 Implement routinely writing exercise prescriptions during ambulatory clinical patient encounters 2.1.4 Classify the billing and coding mechanisms for physical activity promotion and exercise counselling
	2.2 Promote individual and community engagement	2.2.1 Interpret general barriers (including social and economic) to physical activity that are specific to an individual 2.2.2 Describe general barriers (including the built environment, traffic, pollution, and climate) to physical activity that are specific to a community 2.2.3 Organise community resources available for patients to engage in physical activity 2.2.4 Examine the bias (implicit or explicit) that can affect one’s ability to counsel patients
Practical elements		
• As part of a longitudinal experience in residency, write 30 exercise prescriptions (adults and/or children), including both aerobic exercise, resistance training, balance and flexibility, with disease-specific recommendations when indicated • Over the course of residency education, record and then view five clinical encounters of physical activity promotion in 1-to-1 and/or group sessions and examine opportunities for improving content delivery and patient comprehension • Examine physical activity log for 30 patients (approximately 10 per year) • Perform 10 chart audits with a medical coding specialist of patients receiving exercise counselling • As part of a quality improvement initiative, examine the effect of physical activity on one prioritised quality measure (eg, blood pressure, blood sugar, body mass index, depression) within the home institution • Locate at least five different types of accessible resources (eg, community partners, online videos) that can be used to assist physical activity promotion for patients or a community • Identify at least one funding source to assist patients in need of financial assistance to undertake a physical activity programme • Serve as a medical volunteer for a community event focused on physical activity (eg, 5 k run)		
3.Self-care with physical activity	3.1 Understand that self-care can enhance wellbeing, minimise burnout, and improve effectiveness of patient counselling	3.1.1 Demonstrate how to develop a personal wellness plan
Practical elements		
• Create a personal wellness plan with Specific, Measurable, Attainable, Relevant and Time-Based goals that are adopted for at least 1 month, with reviews and modifications every 7 days as needed • Participate in local fitness event (eg, charity run/walk, group fitness class)		

Reproduced from: L. Asif et al. “Exercise medicine and physical activity promotion: core curricula for US medical schools, residencies and sports medicine fellowships: developed by the American Medical Society for Sports Medicine and endorsed by the Canadian Academy of Sport and Exercise.” British Journal of Sports Medicine. 56(7):369–375, 2022.^9^ https://bjsm.bmj.com/content/56/7/369—with permission from the BMJ Publishing Group Ltd.

Before discharge, I reassured my patient that her newly prescribed medications are effective and efforts to eat healthy were beneficial. I assessed her baseline level of PA, then recommended she uptitrate her aerobic activity to 30 min sessions five days a week, with strength training across two of the weekly sessions. I provided education on basic exercises and resources, helping her to set an achievable goal for reassessment in one month[10]. She was excited and motivated to begin. With proper education and specific instructions on how to start and sustain PA, patients leave our care feeling more confident they can improve their health. To improve the health and lives of our patients, it is vital that we integrate PA prescriptions within medical education and clinical practice.

## Data Availability

Data included in the figures and tables were obtained from outside sources with permission from The Creative Commons license as documented in the manuscript.
